# Improving the Mechanical Strength of Dental Applications and Lattice Structures SLM Processed

**DOI:** 10.3390/ma13040905

**Published:** 2020-02-18

**Authors:** Cosmin Cosma, Julia Kessler, Andreas Gebhardt, Ian Campbell, Nicolae Balc

**Affiliations:** 1 Department of Manufacturing Engineering, Technical University of Cluj-Napoca, 400641 Cluj-Napoca, Romania; nicolae.balc@tcm.utcluj.ro; 2 Institute for Toolless Fabrication, 52074 Aachen, Germany; j.kessler@iwf-research.de; 3 Department of Mechanical Engineering and Mechatronics, FH Aachen University of Applied Sciences, 52064 Aachen, Germany; gebhardt@fh-aachen.de; 4 Loughborough Design School, University Loughborough, Loughborough LE11 3TU, Leics, UK; r.i.campbell@lboro.ac.uk

**Keywords:** stainless steel, particle size, particle shape, process parameters, processability index, tensile strength

## Abstract

To manufacture custom medical parts or scaffolds with reduced defects and high mechanical characteristics, new research on optimizing the selective laser melting (SLM) parameters are needed. In this work, a biocompatible powder, 316L stainless steel, is characterized to understand the particle size, distribution, shape and flowability. Examination revealed that the 316L particles are smooth, nearly spherical, their mean diameter is 39.09 μm and just 10% of them hold a diameter less than 21.18 μm. SLM parameters under consideration include laser power up to 200 W, 250–1500 mm/s scanning speed, 80 μm hatch spacing, 35 μm layer thickness and a preheated platform. The effect of these on processability is evaluated. More than 100 samples are SLM-manufactured with different process parameters. The tensile results show that is possible to raise the ultimate tensile strength up to 840 MPa, adapting the SLM parameters for a stable processability, avoiding the technological defects caused by residual stress. Correlating with other recent studies on SLM technology, the tensile strength is 20% improved. To validate the SLM parameters and conditions established, complex bioengineering applications such as dental bridges and macro-porous grafts are SLM-processed, demonstrating the potential to manufacture medical products with increased mechanical resistance made of 316L.

## 1. Introduction

Nowadays, selective laser melting (SLM) is one of the most used additive manufacturing (AM) technologies applied to produce directly metallic prototypes from virtual models. Under an inert atmosphere, this process is capable to fabricate complex bioengineering applications or even cellular scaffolds made of various powders such as titanium alloys [1,2,3], NiTi [4], CoCr [5] or stainless steel [6]. Much available research is developed using 316L stainless steel powder, demonstrating how process parameters can influence the tensile strength, elongation, porosity or microstructure [7,8,9,10,11,12,13]. In recent systematic reviews, a tensile strength up to 620–730 MPa for SLM-manufactured parts made of 316L is defined [14,15]. However, in order to be used by the industry, the SLM medical applications must also have a stable processability, to print parts in good conditions with high resistance.

Due to the low costs of 316L stainless steel compared to titanium or CoCr alloys, it is frequently applied to the manufacture of dental ramus blades, ramus frame implants [16], customized dental crowns or bridges [17], orthopedic prostheses for hip and knee replacements [18], cardiovascular stents [19] and bone tissue engineering scaffolds [6]. In these fields, there is an increasing demand for custom medical parts or lattice grafts. The main defects that can occur in these complex applications are typical for SLM manufacturing such as macro- and microscopic cracks, gas voids, heat-affected zones or a residual stress phenomenon. These technological defects are a consequence of rapid melting and cooling cycles and new research on optimizing the SLM process parameters for 316L powder is needed to limit them.

At the international level, the highest-selling SLM machines to research institutes and companies are limited to 200 W laser power [20]. This aspect emphasizes the compulsion to identify a set of parameters that could be used by these SLM machines. Moreover, to increase the efficiency of SLM manufacturing via reducing the weight of 316L parts and to maintain the same safety factor of products, a high yield tensile strength is demanded.

Different companies are providing 316L powder but full details regarding the particle size, distribution, shape or flowability are not conferred. This information is essential in adequate preparation and controlling of SLM process because at high levels of irradiation energy, the fine particles of 316L can evaporate, generating porosity and decreasing the mechanical characteristics. Commonly, the information offered by powder providers is limited to the mean diameter of particles or the diameter range.

The entire remarks exposed in this introduction motivate the present study. The purpose of this paper is to identify the SLM parameters able to manufacture, in a stable process, medical applications with high tensile strength made of 316L powder. In this work, a commercial powder is analyzed to understand the particle size, distribution, shape and flowability. Process parameters under consideration include laser power up to 200 W, laser scanning speed, hatch spacing and layer thickness. The effect of SLM parameters on processability is evaluated, being concentrated to obtain a stable fabrication and to limit the technological defects. The mechanical tests are developed without applying a stress-relief treatment on samples, only preheating the SLM platform (250 °C) during the fabrication. To validate the process parameters established, complex bioengineering applications such as dental bridges and macro-porous grafts are designed and SLM-manufactured. Due to extended processability investigation, the novelty of this research relates to an increased mechanical resistance of parts made of 316L powder, SLM-manufactured by common solid laser limited to 200 W.

## 2. Experimental Section

### 2.1. AISI 316L Powder

The examinations are carried out using commercially austenitic 316L steel powder. This gas-atomized stainless steel powder was purchased from MCP HEK Tooling (Lübeck, Germany). Its chemical composition is shown in Table 1 (material data sheet). This type of stainless steel is also known as UNS S31603 (North America) or EN 1.4404 (Europe). The extra-low carbon content and increased concentrations of chrome (17%) and nickel (12%) enhance the durability against corrosion. Moreover, 316L demonstrates a medium biocompatibility in vitro and in vivo [21,22].

### 2.2. Particle Analysis

A typical instrument for particle characterization is Morphologi G3 system (Malvern, UK). Using this instrument, the powder was dispersed with an instantaneous pulse of compressed air and precise measurements were elaborated [23]. The diameter and shape of the particles were measured, as well as counting the number of them, in order to get the distribution, according to ISO 13320 [24]. In this study, 46,475 particles were counted at 15 mm^3^, at 4-bar pressure using 10× optical system magnification (1.75 μm minimum particle size and 100 μm maximum). The particle size distribution was calculated based on two distinct techniques: number distribution of particles and volume or mass distribution. Laser diffraction results were reported on a volume basis, so the volume mean was used to define the central point [25]. The particle size distribution is described in percentiles: d10, d50 and d90. These d-values indicate that 10%, 50% and 90% of particles are finer than this diameter. Additionally, the volume mean diameter D (4,3) and the mean value of surface area distribution D (3,2) were estimated with Equations (1) and (2).

(1) $$D(4,3)=\frac{\sum_{1}^{n}D_{{}_{i}v_{i}}^{4}}{\sum_{1}^{n}D_{{}_{i}v_{i}}^{3}}$$

(2) $$D(3,2)=\frac{\sum_{1}^{n}D_{{}_{i}v_{i}}^{3}}{\sum_{1}^{n}D_{{}_{i}v_{i}}^{2}}$$

The particle shape was analyzed by circularity, convexity and elongation parameters. Each parameter is described below (Section 3.1). In the field of statistics, standard deviations (STDV) and relative standard deviations (RSD) calculations are preferred. The particle flowability was measured using a hall flowmeter funnel with an orifice of 2.5 mm and calculating the flow rate (s/50 g). The hall flow rate was expressed as the time required for a 50 g powder sample to be discharged by gravitational force through the flowmeter funnel (ASTM B213 [26]).

### 2.3. Manufacturing Conditions

**Figure 1:**
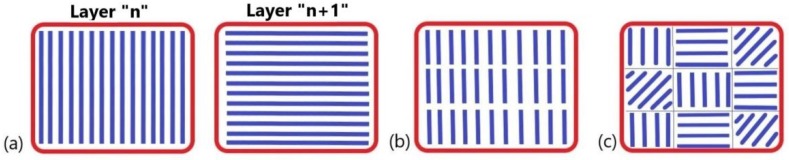
Laser scanning strategies: (**a**) X/Y alternating scanning; (**b**) Stripe hatch pattern; (**c**) Chessboard hatch pattern (or islands).

A SLM Realizer 250 machine (Realizer GmbH, Borchen, Germany), equipped with a 200 W solid fiber laser (type Nd:YAG) was used to manufacture the samples. The laser scanning strategy adopted was X/Y which allows a scanning in X direction of “n” layer and in Y direction of “n + 1” layer (rotating each hatch scan 90°). The advantage of this hatch pattern is that it can contribute to a high build rate compared with stripe hatch or chessboard pattern (see Figure 1). On the other side, this scanning strategy can cause a significant residual stress phenomena compared to the chessboard hatch pattern [27,28], and a special attention was given to process parameters.

The 316L samples were SLM-manufactured, varying the processing parameters such as laser power (50–200 W) and scanning speed (250–1500 mm/s). The hatch space and layer thickness were 80 μm and 35 μm, in concordance with the literature [11]. To program the scanning speed in REditor software (Version 1.1, Realizer GmbH, Borchen, Germany), two variables were defined as following point distance and exposure time. Thus, the scanning speed was estimated using Equation (3). The density energy (*E*) was calculated by Equation (4):

(3) $$v=\frac{\mathrm{point}\;\mathrm{distance}}{\mathrm{exposure}\;\mathrm{time}}$$

(4) $$E=\frac{P}{v*h*t}\left(J/{\mathrm{mm}}^{3}\right)$$

where *P* is the laser power (W), *v* is the scan speed (mm/s), *h* is the hatch spacing (mm) and *t* is the layer thickness (mm).

**Figure 2:**
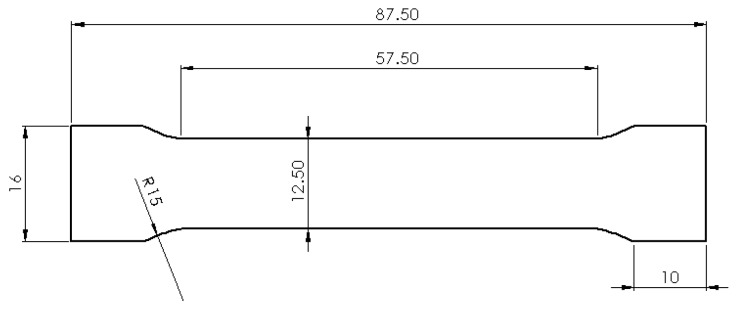
Dimensions of a standard sample for tensile testing at room temperature.

The design of samples was done in concordance with ISO 6892 [29] and is presented in Figure 2. The orientation of these tensile specimens on SLM platform is horizontal as was suggested by previous studies [9,13]. The design of supports include block structures with 2 mm distance. The tooth base interval and length was 2 mm, respectively, and the tooth top length was 1 mm. These configuration of support structures were used to anchor and sustain the samples. They were modelled in Magics software (Version 13, Materialise, Leuven, Belgium) and may limit the warps and cracks of parts [11]. To test different laser parameters on the SLM plate, a soft recoater made of dense carbon fibers with Ø 7.5 μm was used to spread the powder. This flexible recoater allows a continuation of the process when the metal was distorted and protruded out of the powder bed (e.g., small peaks). Hence, a collision which can de-calibrate the powder bed thickness was avoided. This technological issue cannot be managed by a hard recoater made of a stainless steel blade.

The SLM platform was uniformly preheated at 250 °C during the fabrication. Under a high-purity Ar-atmosphere and limited oxygen level (0.3–0.5%), the samples were manufactured with different SLM process parameters and the working conditions detailed above.

### 2.4. Processability Investigation

The SLM technology can produce a residual stress phenomenon caused by temperature gradients. In the laser beam spot, temperatures above the melting point prevail, while the rest of the part cools rapidly [28]. Depending on the part geometry and powder material type, the temperature gradient can lead to several defects such as cracks, severe warping, distortion, spatter or a heat-affected zone. To reduce the impact of these unfavorable thermal stresses, processability evaluation and optimization is required. The achievement of such a practical target was possible by surveying the manufacturing process and compiling a scientific report for each set of laser parameters. A comprehensive report addressed all the technological defects, as well as the interventions and distortions that occurred during the fabrication. In order to quantify these observations, the following processability ranks were defined:

1. Unstable processability (U): characterizes the impossibility of finalizing the process due to high residual stresses that severe warping the part, being necessary to interrupt its manufacturing. Major risk of failing.
2. Relatively stable processability (R): characterizes a medium chance to succeed the process with continuous surveying until the last layer is deposed, being possible to observe some limited macro-cracks (0.1–1 mm length) or heat-affected zones.
3. Stable processability (S): characterizes a successful SLM process, which does not need continuous surveillance. The parts obtained do not present the defects mentioned above.

### 2.5. Physical-Mechanical Testing

Tensile stress–strain behavior was measured using standard sheet specimens in a universal testing system type Zwick Z100 (ZwickRoell, Ulm, Germany). The design of standard specimens is presented in Figure 2. The mechanical tests were performed according to ISO 6892 [29]. Working parameters of Zwick equipment were 2 mm/min load rate and 50% humidity at room temperature (18 °C). After post-processing the samples by removing the support structures and cleaning the surfaces, the initial cross section of specimens was approximately 37.50 mm. The post-processing was performed by sandblasting the surfaces with fine alumina grains (size of 120 μm) at a pressure of 5 bars. The surfaces with supports were finished by milling using a pneumatic spindle. The tensile samples in as-fabricated conditions were tested for their mechanical characteristics such as yield strength (YS), ultimate tensile strength (UTS), Young modulus and elongation at fracture. The relative density of SLM samples was determined using an analytical balance (Partner AS 160.R2, precision ± 0.2 mg, Radwag, Miami, FL, USA), based on Archimedes’ principle and taking into consideration 8 g/cm^3^ as a reference value for 316L stainless steel.

### 2.6. Dental Bride and Lattice Scaffolds Design

**Figure 3:**
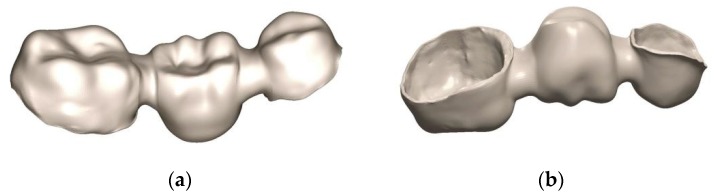
Complex customized dental bridge with thin walls: (**a**) Isometric view; (**b**) Inner walls.

To test the results on medical applications, some complex models were designed. The first model is a customized dental bridge, containing multiple anatomical shapes, sharp internal corners and uneven thicknesses (Figure 3). These geometrical characteristics could induce residual stress because the thicker regions cool down slower than thinner walls and can distort the dental bridge. The main steps to design the dental bridge are specific to the restorative dentistry field [30,31]. Initially, an intraoral digital scan of a prepared mandibular premolar (35) and molar (37) was conducted. The Dental Wings IntraOral (DWIO, Montréal, QC, Canada) system was use to capture multi-scan imaging with a continuous blue light projection. The resulting model was imported into DWOS (Dental Wings) software and the 3D metallic substructure of a dental bridge was designed with 0.50 mm wall thickness. This part was orientated horizontally on the SLM platform and it was anchored with the support structures presented above (Section 2.3).

**Figure 4:**
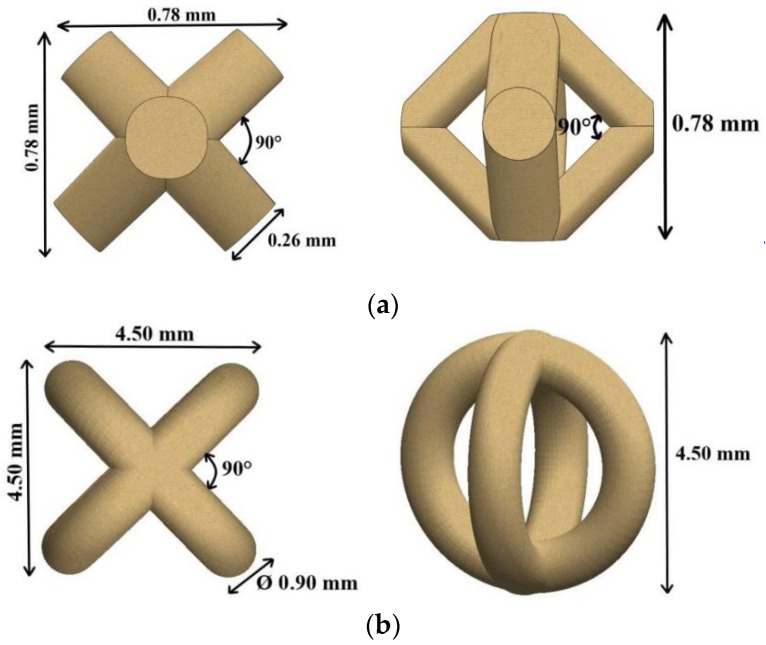
Basic unit cell, top and front views: (**a**) Body-Centered Cubic (BCC); (**b**) Circles Intersection (CI).

The second and the third models were elaborated considering the state-of-the-art of SLM processing which suggests that lattice structures from 316L hold great promise for the fabrication of patient-specific implants via bone tissue engineering [6]. These macro-porous grafts made of metallic biomaterials act as a template for ingrowth osseointegration and bone tissue formation. In general, the SLM scaffolds can mimic the porosity and functions of a native human tissue. The present grafts were designed in Creo Parametric software (Version 5.0, PTC, Boston, MA, USA). The virtual models of lattice units and their main features are shown in Figure 4. These basic unit cells were multiplied periodically and were uniform in cylinders with 15 mm height and 10 mm diameter. These lattice structures were conceived in accordance with bone tissue engineering requirements. The macro-porosity designed was 60% for Body-Centered Cubic (BCC) specimens and 80% for Circles Intersection (CI) grafts. Five prototypes were manufactured for each model using the laser parameters and SLM conditions established for a stable processability and high tensile strength. These specimens were investigated by scanning electron microscopy (SEM). The morphological characteristics of the surfaces were recorded and the accelerating voltage set up is mentioned in the footnote of pictures. The real porosity of lattice structures was determined through Archimedes’ method [32].

## 3. Results and Discussion

### 3.1. Powder Examination

**Figure 5:**
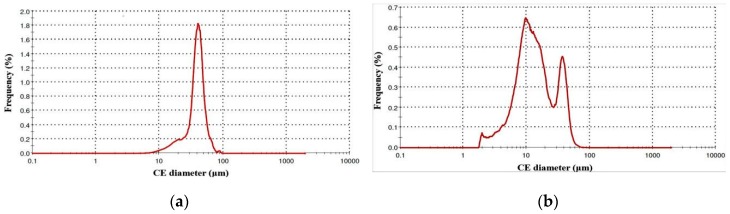
Diameter report of 316L particles: (**a**) Volume distribution method; (**b**) Number distribution method.

Figure 5 presents the distribution graphs based on frequency curves and Table 2 summarizes the diameter reports. The Circle Equivalent (CE) diameter value shown is the diameter of a circle with the same area as the 2D image of the particle. Figure 5a shows the granulometry chart regarding the diameter of 316L particles elaborated by volume distribution method. In general, this is the most used method to determine the diameter mean of particles in powder feedstock. The volume size distribution follows a Gaussian curve with a mean diameter D (4,3) of 39.09 μm (Table 2). The mean value of surface area distribution D (3,2) was estimated at 33.67 μm. From a volume distribution point of view, 50% of particles have a diameter less than 39.34 μm (d50) and 10% are finer than 21.18 μm (d90).

In Figure 5b is shown the diameter chart of 316L particles via number distribution method, showing that the diameter of the particles varied from 1.90 to 91.28 μm. The numbered fine particles with diameters up to 12.80 μm represent 50% (d50) of the total accounted grains (Table 2). These fine particles can have a significant effect on the SLM samples because they are melting rapidly, which is useful, but in the same time they can also evaporate, generating porosity and decreasing the mechanical characteristics. In this case, the information delivered by the powder producer was limited to the diameter range of particles (20–50 μm). Therefore, the particle size and distribution were examined. It was observed that in this 316L powder, the numbered fine grains have a diameter between 5–12 μm and they are less than 10% of the volume feedstock.

Chen et al. recently investigated the microstructure and mechanical properties of 316L samples and found that even if a fine powder (d50 = 8.2 μm) or a normal one (d50 = 20.3 μm) is used, the tensile strength will be closer after SLM manufacturing with 200 W laser power [33]. This indicates that the ultimate tensile strength was 611 MPa for samples SLM-processed from fine 316L powder or 597 MPa when the normal powder was tested [33]. Because the particle size and distribution do not significantly modify the tensile strength, the correlation of SLM process parameters on powders such as the present one was essential. Knowing that the particle diameters can increase with number of build runs and sieving procedures [34], it can explain why we found particles with diameters up to 91 μm. The actual 316L steel powder was recycled five times and the presence of large grains in powder feedstock is limited, because 90% of particles have a diameter less than 52 μm (Table 2, d90). During the fabrication, the layer thickness was set at 35 μm and large particles could not be present in the powder bed. The knowledge given by this powder examination, focused on granulometry variation, is important since it can influence the SLM processability throughout the entire manufacturing build-up.

**Figure 6:**
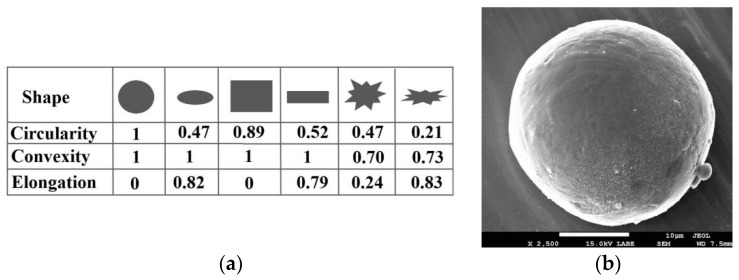
(**a**) Shape parameters used to investigate the 316L particles and their value significance; (**b**) SEM image of 316L particle.

**Figure 7:**
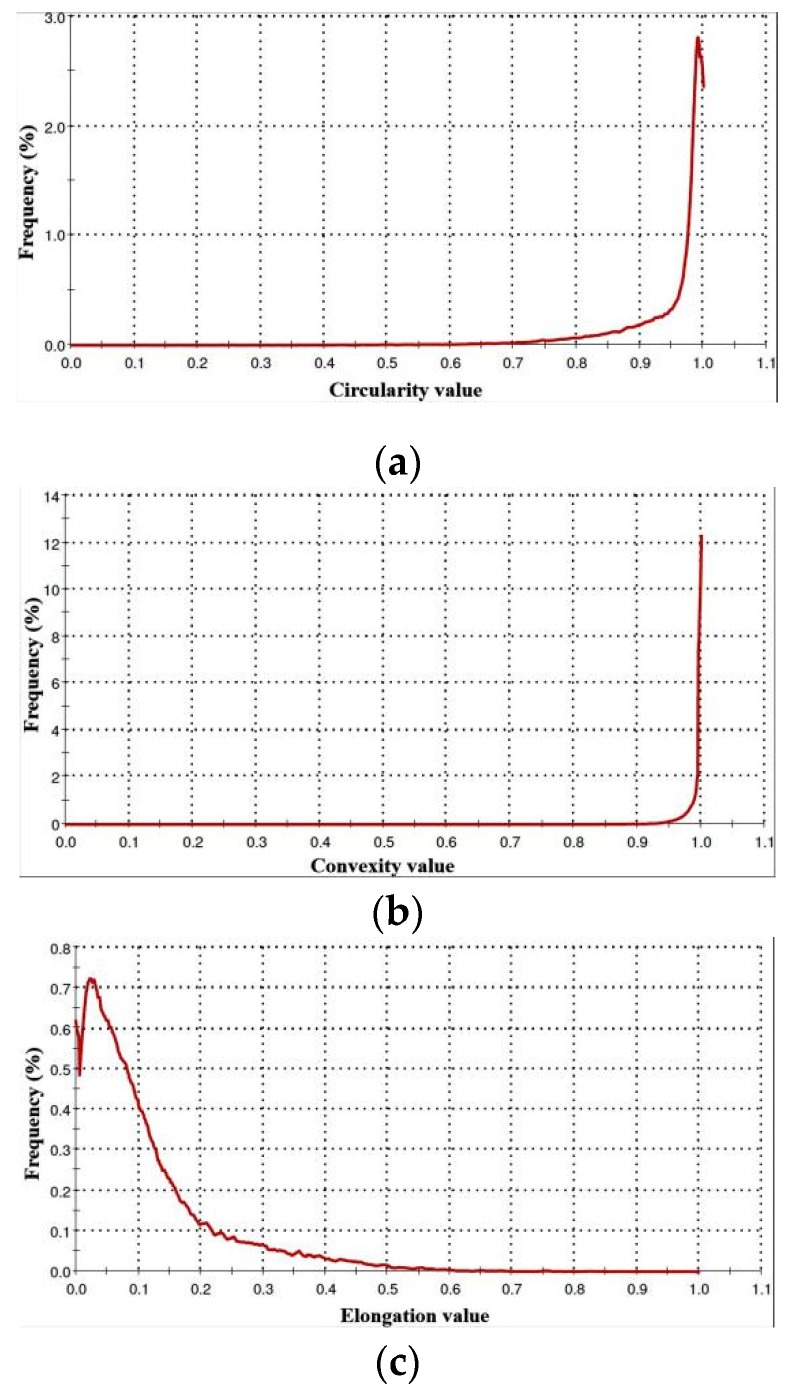
Particle shape charts: (**a**) Circularity; (**b**) Convexity; (**c**) Elongation.

To provide a quick and easy comparability, the significance of shape parameters is illustrated Figure 6a. A SEM characterization of a 316L particle is shown in Figure 6b. The reports of shape parameters are detailed in Table 3 and Figure 7 (circularity, convexity and elongation). Each parameter is scaled between 0 and 1, in concordance with frequency.

Figure 7a presents the high sensitivity circularity chart, suggesting that in general, the particle shape is similar to an ideal circle, having a mean value of 0.94. Here, the 0 value means the most oblong grains. Because this parameter is dedicated to analyzing the modifications of overall form, it was also observed that 10% of particles have 0.83 circularity value (d10). The circumference of a few particles could be close to an ellipsoid.

As shown in Figure 7b, 90% of the total investigated particles lie within 0.97 ± 0.02 convexity value (reversed of d10). Thus, 0 value means an abstract form with multiple sharp edges and points, and 1 means a perfectly round and smooth surface. This shape parameter evaluates the surface roughness changes. Analyzing it, we identify that, predominantly, the particles have a reduced surface roughness. This low surface roughness of particles is also shown in Figure 6b.

Since the elongation parameter measures the length–width relationship, Figure 7c shows that the mean value calculated is 0.11, suggesting that the aspect ratio is similar to a smooth ellipse (see Table 3). The present examination of 316L powder certifies that the majority of particles are nearly spherical with a volume mean diameter of 39.09 μm ± 12.23 μm, and 50% of them hold a diameter less than 39.34 μm (d50).

The hall flow rate of this 316L powder was 18.2 s per 50 g. This is a high flowability compared with other 316L powders (e.g., LPW 316 [35]). This can be attributed to the fact that the powder did not have a wide particle size distribution with a large amount of small particles and it also has a high convexity parameter (described in Table 3). The small particles are less than 10% of the total volume of powder and their diameter is up to 21 μm (Table 2, d10 value from volume method). Due to a reduced percentage of fine particles, they did not influence the flow rate of 316L powder. According to ASTM B213 standard [26], the fine powders may not flow. Moreover, the convexity shape parameter evaluates the surface roughness of grains and its value is approximately 1, meaning that the interparticle friction is limited, improving the flowability. The ability of a powder to flow is a function of interparticle friction. As a general rule in the SLM process, a powder with high flowability tends to guarantee reproducible deposition of single powder layers, higher powder packing density and better mechanical properties [35].

### 3.2. Manufacturing Evaluation

**Figure 8:**
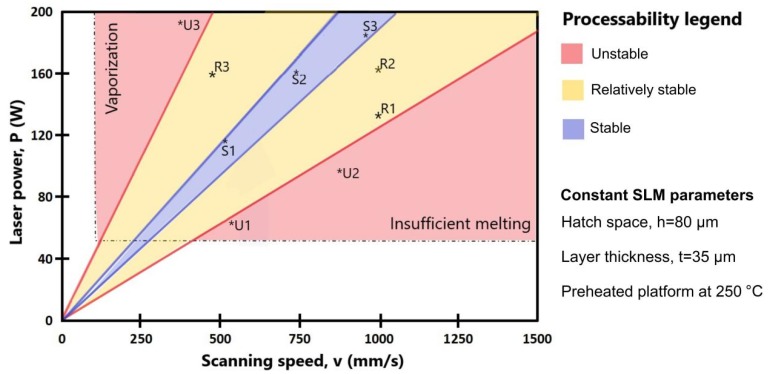
Processability map of 316L powder via selective laser melting (SLM) technology; some representative laser configurations are marked for each processability index.

Analyzing the literature recommendations regarding the SLM process parameters which could be proper for 316L powder [7,8,9,14,15,33,36] and based on our previous studies [3,31,37,38], different combinations of processing parameters were configured, tested and inspected. On each SLM platform more than ten samples were processed in the same time with distinct laser parameters and the carbon fiber recoater helped to continue the jobs when metal distortions appeared without affecting the other samples. In total, more than 100 samples were manufactured with various process parameters to define a map of processability. Figure 8 summarizes the effort undertaken to identify some sets of SLM parameters for stable processability on a preheated platform at 250 °C. As it was reported previously [13], this study also confirms that a uniform preheating significantly decreased the manufacturing defects and it can provide a relatively stable or stable processability.

**Figure 9:**
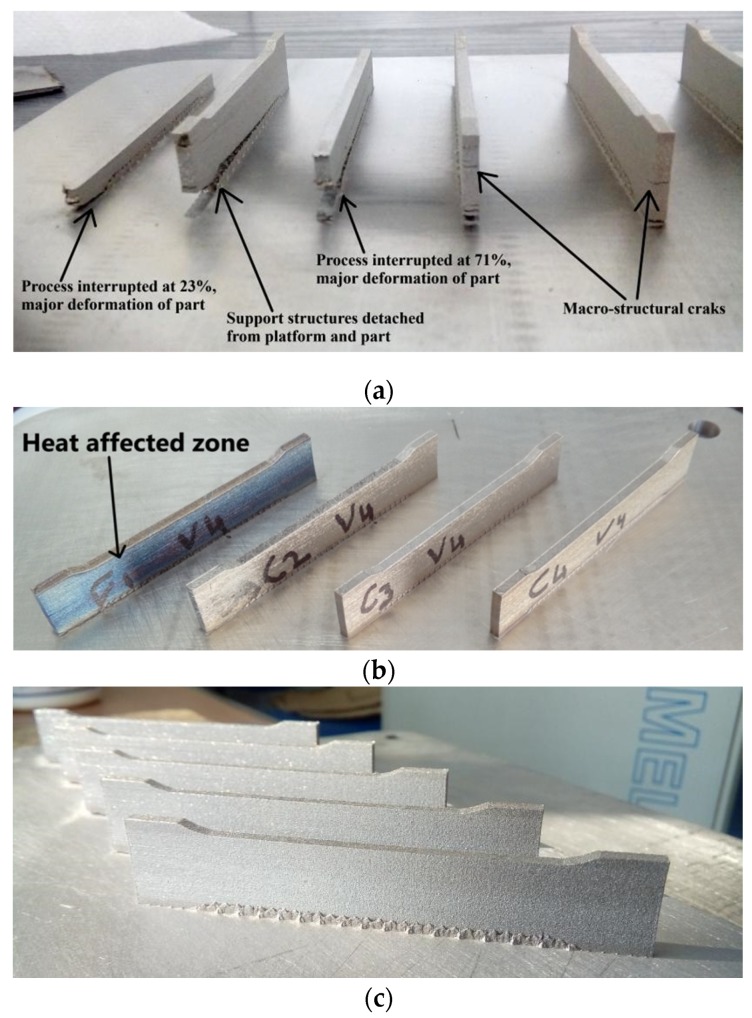
Effect of different parameters on processability: (**a**) Unstable processability and defects generated; (**b**) Part with heat-affected zone, relatively stable processability (energy density of 142 J/mm^3^, sample R3); (**c**) Stable processability using proper SLM parameters, free of cracks or warping (sample S3).

In the context of manufacturing evaluation, understanding the effect of laser parameters is a complex issue itself. An unstable process was observed in more than 30 sets of parameters. Some of them are marked in red areas from Figure 8 and they are as follows: U1 (60 W, 550 mm/s), U2 (95 W, 800 mm/s) and U3 (190 W, 400 mm/s). The main defects observed for unstable processability were cracks, severe warping, distortion and vaporization. These defects break or interrupt the manufacturing and some examples are shown in Figure 9a. They were generated by residual stress accumulated in samples, affecting considerably the SLM processability and causing a premature failure in manufacturing. Previous studies also reported similar defects and issues, being explained by temperature gradient mechanism [15,34,39]. Based on this metallurgical behavior, pre-solidified material underneath the melted layer is heated up rapidly upon laser irradiation, which readily expands, but is constricted by the cold and rigid portions of the solidified piece, repeated in multiple cycles [40,41]. It was also observed that when the samples were rotated around the Z axis by 45° (e.g., Figure 9b), the recoater does not suddenly meet a long section, reducing the risks of crashes. In this manner, the laser melts fractions of hatch region because they were angled at 45° and the laser movement is parallel to X or Y axes. The scanning area was not continuously melted, limiting the effect of heat-affected zones. The present processability evaluation suggests that a relatively stable SLM manufacturing can be expected using the parameters attributed to yellow area from Figure 8. Samples fabricated in a relatively stable processability can be seen in Figure 9b, one of them being exposed to the heat-affecting zone phenomenon. A stable melting was obtained when working with an input energy between 65–80 J/mm^3^. For a stable processability, the variation of laser parameters is more restricted as can be seen in Figure 8 (blue area). In Figure 9c are illustrated samples printed in a stable processability, significantly limiting the main manufacturing defects detailed above. Some laser parameters for stable or relatively stable processability can be seen in Table 4.

The scanning strategy configured also plays a significant role in limiting the internal stress. Because our study is focused on the effect of process parameters on SLM manufacturing, the scanning strategy adopted was X/Y alternating scanning at 90°. This hatch pattern contributes to a high build rate but it could cause a significant residual stress phenomenon [27,28]. Nevertheless, the results reported recently contradicted the popular conception of dividing the scan area into small regions being an effective scanning strategy for residual stress reduction and 90° alternating scanning strategy resulted in the lowest residual stress build-up for SLM parts [42]. From our technological observations, combining the 90° alternating scanning with proper SLM process parameters can induce a stable SLM manufacturing of 316L parts. Moreover, the layer thickness is essential in obtaining a stable SLM processing.

### 3.3. Physical-Mechanical Properties

**Figure 10:**
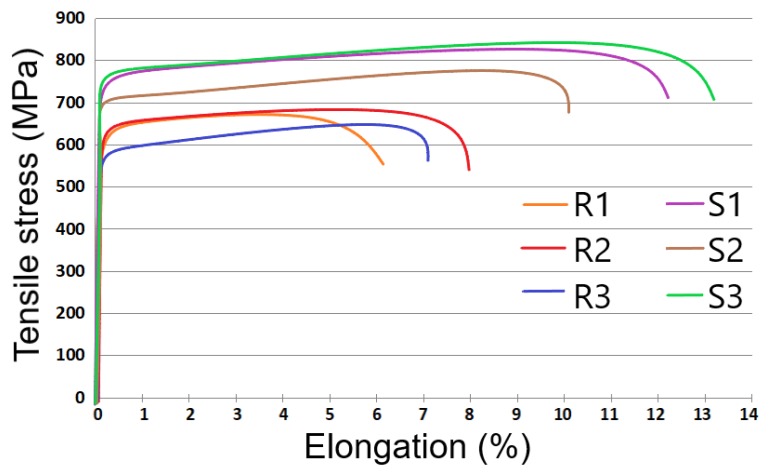
Representative tensile stress vs. elongation plots obtained through mechanical tests on SLM-manufactured samples (relatively and stable processability).

Besides providing a stable processability, investigating the effect of SLM parameters on mechanical characteristics is compulsory. The tensile tests were carried out on samples processed in relatively stable or stable processability and the SLM parameters are detailed in Table 4. The samples were printed horizontally on the SLM platform, as can be seen in Figure 9c. Representative tensile stress vs. elongation responses of samples are displayed in Figure 10. Tensile properties such as yield strength (YS), ultimate tensile strength (UTS), elongation at fracture and Young modulus are listed in Table 4. Their relative density is also presented. The tensile tests showed the anisotropic behavior of SLM samples due to the generative production principle also observed in other studies [9,43].

From our findings, the YS can vary between 590–780 MPa, the UTS from 640 MPa to 840 MPa and the Young modulus between 173–194 GPa (±3% standard deviations). The highest tensile strength was obtained on samples manufactured via process parameters with codes S1 and S3 shown in Table 4. This superior mechanical resistance can be attributed to the fine crystalline structure created by the rapid solidification during the process of building the material in thin, discrete layers [12,15].

Even if various studies suggest that a density energy of 104 J/mm^3^ can increase the tensile strength of 316L parts [8,34], we observed that even a lower one (67 J/mm^3^ belongs to S3, Figure 10) and a stable processing can also conduct improved YS and UTS. Furthermore, recent compressive research reported that an input energy between 60–70 J/mm^3^ can raise the UTS up to 700 MPa [44].

Additionally, a preheating over 200 °C during the SLM fabrication led to a dense homogeneous structure with low porosity, supporting moreover the growing of YS and UTS properties [13]. As it was reported, even the protective gas can play a significant role in obtaining a high relative density if argon is used instead of nitrogen, deoxidiser or helium [13]. The actual study was developed on a preheated SLM platform at 250 °C and using pure argon. The oxidation of 316L powder was limited because the oxygen content in the working chamber was 0.3–0.5%. All these manufacturing conditions increase the relative density of SLM samples over 99% and the tensile strength (see Table 4).

The highest values of YS, UTS and elongation at fracture obtained in different studies published in the period of 2008–2019 are summarized in Table 5. These studies were conducted using 316L powder with similar particle size to ours. Each study demonstrates the importance of SLM parameters in mechanical behavior of parts. Until now, the YS range was 430–640 MPa and the UTS varied from 480 to 745 MPa. In this study, the YS and UTS are higher compared to other disseminated research (Table 5). From a laser power point of view, recent works elaborated with high laser powers (300–1000 W) reported a YS between 510–580 MPa and a UTS between 620–730 MPa [8,44]. Moreover, Luo et al. demonstrated that a laser power between 170–200 W can achieve a tensile strength similar to that obtained using 290 W, reporting an UTS of approximately 700 MPa [14]. The present results also show that is possible to raise the tensile properties higher if a common SLM system with a laser limited to 200 W is used, adapting the other manufacturing parameters and conditions. This fact is discrepant with popular conception, which suggests that just a high laser power can induce an improvement of mechanical characteristics. In essence, SLM parts are often produced with lower ductility and anisotropic behavior depending on process parameters and other manufacturing conditions [34]. Analyzing the results described by Mower et al. and Yakout et al. which investigated the mechanical properties of 316L samples fabricated by a similar SLM technology (direct metal laser sintering, EOS, Krailling, Germany), the YS and UTS obtained were lower than the present results (e.g., YS between 496-510 MPa and UTS between 700–717 MPa) [12,44].

Correlating with other laser techniques with the same principles of operation, such as Laser Engineered Net Shaping (LENS) [43], the tensile strength obtained in the present work is 10–20% improved. On the other hand, the 316L LENS samples demonstrate a significantly high value of elongation up to 46%. Compared to conventional casting and annealing sheets or welded joints made of 316L, the present YS is two times higher (see Table 5). As reported by Kurzynowski et al., the specific microstructure of austenite 316L SLM samples in as-built conditions is the cause of a double increase of yield strength and significant reduction of elongation at fracture in comparison with properties of conventional casting and annealing of 316L sheets [7].

Regarding the elastic modulus, the values determined are in concordance with the literature [11,12,13]. The 12–13% elongation at fracture of SLM samples is similar to what has been reported [13,33,35]. However, the elongation is not satisfactory and is still below the standard, which requires a minimum of 40% (details in Table 5). To improve the elongation at fracture, future heat treatments or hot isostatic pressing are demanded on 316L parts.

### 3.4. Validating the Knowledge

**Figure 11:**
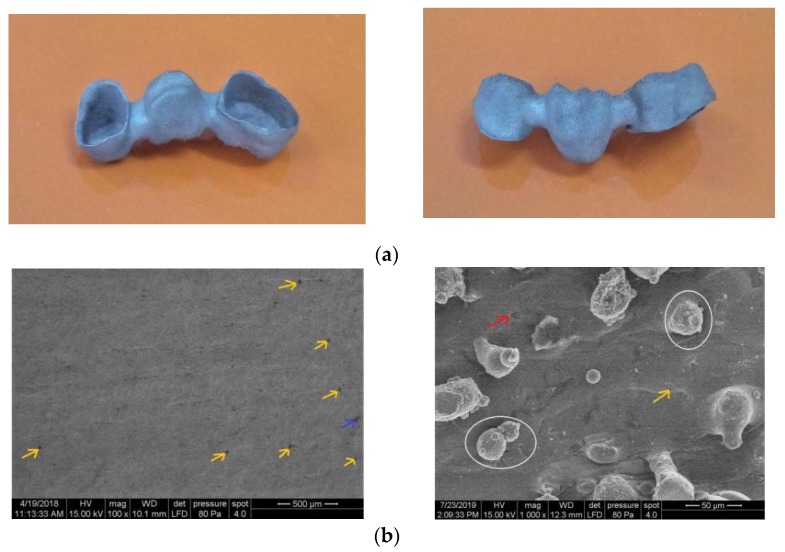
(**a**) SLM-manufactured dental bridge using optimal process parameters established for 316L powder; (**b**) SEM images of surface morphology (×100 and ×1000 magnification).

**Figure 12:**
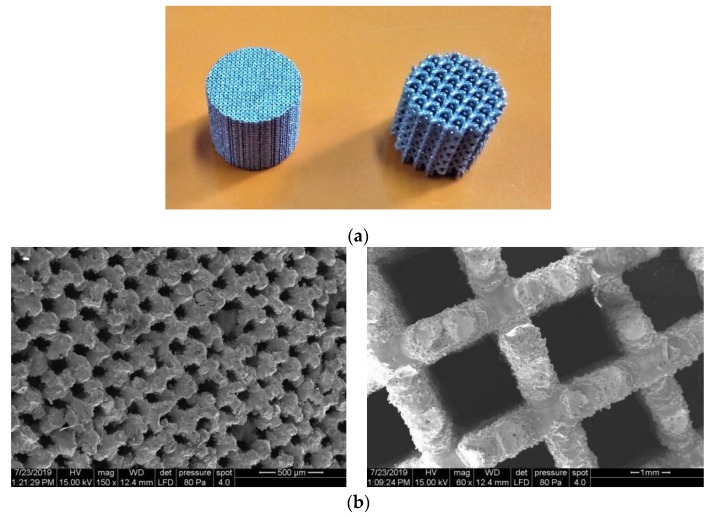
(**a**) Macro-porous scaffolds SLM-manufactured using 316L powder; (**b**) SEM images of BCC structure (**left image**) and CI structure (**right image**).

Even if pure titanium or its alloys are often practiced in medical sectors due to good integration into the human body, in particular cases, 316L stainless steel is used for certain applications such as in dental crowns, brides or implants, orthopaedic prostheses and bone tissue engineering scaffolds [6,18]. This observation was taken into account for manufacturing cases. To validate the SLM parameters and conditions proper for a stable processability and high mechanical strength, five dental bridges and ten scaffolds were fabricated from 316L powder (Figure 11 and Figure 12). The SLM parameters and conditions used to build up these parts are: 180 W laser power, 950 mm/s scanning speed, 80 μm hatch space, 35 μm layer thickness, X/Y alternating scanning strategy, preheated platform at 250 °C and low oxygen level during fabrication (<0.5%) in an Ar-atmosphere environment (details in Table 4, code S3). Due to the high melting speed and scanning strategy adopted, the production time was reduced. In order to avoid the need of supports in cavities, the dental bridges were built horizontal with orifices up.

A printed dental bridge is shown in Figure 11a. The vertical wall was investigated by SEM. It can be seen that the surface has limited defects and it is homogeneous (Figure 11b). Some un-melted powders and laser spatter particles are sticking on 316L surface. This surface morphology was also reported previously [33]. The laser spatter particles originates as a consequence of the complex dynamics taking place in the melt pool, having spherical morphology and an austenitic phase similar to starting 316L powder [46]. A few laser spatter particles are indicated in Figure 11b (in white circles).

A cavity associated with a binding defect is indicated by the blue arrow (Figure 11). Regarding the microstructural defects, a small number of gas micro-pores with 5–25 μm dimension can be observed in Figure 11b (yellow arrows). These spherical voids are associated with gas entrapment [15]. Micro-cracks are recorded randomly on the 316L surface, having a maximum 20 μm length (red arrow). They appear because under the action of a moving high-energy laser, the melting and solidification processes are completed in a considerably short period of time, which induces a high-temperature gradient and a high stress, as a result of which cracks tend to be formed in order to release the thermal stress [11].

The macro-porous scaffolds were processed vertically as can be seen in Figure 12a. After the lattice samples were cleaned, the final porosity was 57% and 78%. These values are proximate to virtual estimation (60% and 80%). Figure 12b shows SEM images of the BCC and CI struts (cross-sectional view at 7.5 mm from top surface). The strut surface morphology is not cleaner, having a medium surface roughness and attached unmelted particles are visible. A large amount of balls are observed on the strut surface, being a negative phenomenon. To limit this phenomenon, a chemical etching should be considered [47]. Due to these interconnected pores, the scaffold specimens have unique mechanical properties. As previously explained [48], the macro-porosity of parts directly influences their mechanical characteristics. Using a Gibson–Ashby mathematical model, the tensile strength of 316L scaffolds could be predicted depending on the tensile strength of fully dense samples determined and presented in Table 4. Knowing the mechanical properties of strut, finite element analysis could be develop to simulate the behavior of 316L scaffolds. In this way, the engineering stress–strain diagrams can be correlated with theoretical predictions of 316L scaffolds [49]. Future studies are required to evaluate the strut accuracy and imperfections by X-ray microtomography (micro-CT scanning) [50]. To reduce the surface roughness, new post-processing methods should be developed and adapted to enhance the cell activity [51]. Moreover, the scaffold-implant concept can help and give hope to thousands of people affected by various bone diseases, often resulting in partial or full loss of tissues [52,53].

Applying this new generation of grafts in medical applications, the osseointegration process could be improved due to their macro-porosity. The advantages of these 316L scaffolds include saving material consumption, reducing the energy used and diminishing the manufacturing time. From our perspective, the SLM process will completely change the biomedical manufacturing industry, making it much faster, more flexible and customized. Due to the high resistance of SLM parts, they will generate benefits in terms of total production cost, making this emerging technology more adopted by companies.

## 4. Conclusions

The present examination of 316L stainless steel powder demonstrates that most of the particles are nearly spherical with a volume mean diameter of 39.09 μm, and 10% of them have a diameter less than 21 μm (d10). A high flowability of powder was measured, being a consequence of limited interparticle friction. The SLM process parameters identified to alternating melting strategies limit significantly the defects generated by internal stress, even if it is known that the 90° alternating laser scanning may increase the residual stress compared to the chessboard pattern strategy.

The experimental results show that the processability of 316L powder can significantly influence the mechanical properties. A laser power up to 180 W and adaptation of the other SLM parameters can improve the tensile strength and a relative density above 99% can be reached. The SLM conditions and laser parameters established raise the yield strength to 780 MPa and the tensile strength to 840 MPa. Besides the laser parameters, cumulative factors contributed to this result such as: reduced percentage of fine or larger particles in powder feedstock, high flowability of powder, preheating of the SLM platform at 250 °C, pure argon as protective gas, low oxygen level on SLM chamber (< 0.5%), horizontal build-up of samples, and flexible recoater made of carbon fibers. In order to fully understand the mechanical behavior obtained, future metallographic and X-ray diffractions will be developed to analyze the crystal phase of 316L samples produced with these SLM parameters. The SLM parameters and process conditions could be suitable to produce medical parts and lattice structures, in a stable manufacturing environment and avoiding major solidification defects. The yield tensile strength obtained could provide new opportunities for biomedical scientists to reduce the weight of implants, prostheses or cellular grafts made of 316L, while maintaining the same safety factor.

## Figures and Tables

**Table 1:** Chemical composition of the 316L stainless steel powder.

Chemical Element	Cr	Ni	Si	Mn	Mo	P	S	C	O	Fe
**Maximum weight (%)**	17	12	0.75	2	2.5	0.045	0.03	0.03	0.13	Balance

**Table 2:** Diameter report of 316L particles.

Value	Volume Method (μm)	Number Method (μm)
Minimum	1.90	
Maximum	91.28	
Mean	39.09	17.24
d_10_	21.18	5.67
d_50_	39.34	12.80
d_90_	52.12	37.39
STDV	12.23	

**Table 3:** Powder examination via shape parameters.

Value	Circularity	Convexity	Elongation
Minimum	0.06	0.44	0.00
Maximum	1.00	1.00	0.94
Mean	0.94	0.99	0.11
d_10_	0.83	0.97	0.01
d_50_	0.97	0.99	0.07
d_90_	0.99	1.00	0.27
STDV	0.08	0.02	0.11
RSD (%)	9.46	2.04	97.35

**Table 4:** Tensile test results of SLM-processed 316L samples.

Processability Rank		Laser Power (W)	Scanning Speed (mm/s)	Energy Density (J/mm^3^)	YS (MPa)	UTS (MPa)	Elongation at Fracture (%)	Young Modulus (GPa)	Relative Density (ρ_rel_)
Relatively stable	R1	130	1034	44.98	630	672	6	182	98.3
	R2	170		58.82	654	689	8	173	98.5
	R3	160	400	142.85	590	648	7	178	99.6
Stable	S1	110	500	78.57	774	823	12	192	99.3
	S2	150	750	71.42	703	786	10	184	99.3
	S3	180	950	67.66	783	841	13	194	99.1

**Table 5:** Comparison regarding the obtained mechanical properties of SLM-manufactured samples from 316L powder in as-build conditions.

Particle Size (μm)	Laser Power (W)	Scanning Speed (mm/s)	Layer Thickness (μm)	YS (MPa)	UTS (MPa)	Elongation at Fracture (%)	References/Year
d_50_ = 39.3	110	500	35	774	823	12	This study (code S1)
	180	850		783	841	13	This study (code S3)
d_50_ = 37.2	140–290	800	30	N/A	630–730	35–60	[14]/2018
d_50_ = 20.3	200	2000	30	498	589	11	[33]/2018
d_50_ = 27.0	100	300	50	N/A	501–630	11	[13]/2013
d_50_ = 29.1	200–300	600–1000	40	470–510	620–690	15–60	[44]*/2019
22	90	160–640	50	642–643	714–745	15–28	[9]/2008
20–63	200	200	50	517	687	32	[7]/2018
20–63	380–950	625–3000	50	510–580	620–700	30–50	[8]**/2018
15–45	150	700	20	510	620	43	[15]/2017
20–50	90	1000	30	430–530	480–640	12–24	[35]/2017
15–45	195	750	40	496	717	30	[12] */2015
Laser Engineered Net Shaping (LENS)				470–580	700–776	33–46	[43]/2016
Conventional casting and annealing				304	560	60	[45]
Welded joints				290	520	70	[45]
AISI 316L or EN 1.4432				170	485	40	Standard

* Samples processed by similar SLM technology (direct metal laser sintering). ** The energy density was maintained at ~104 J/mm^3^ for all the samples fabricated. N/A indicates that the value is not available.
